# Characterization of tumor remnants in intraoperative MRI-assisted microscopic and endoscopic transsphenoidal resection of less invasive pituitary adenomas

**DOI:** 10.1007/s10143-021-01705-z

**Published:** 2021-12-02

**Authors:** Andrej Paľa, Gwendolin Etzrodt-Walter, Georg Karpel-Massler, Maria Teresa Pedro, Benjamin Mayer, Jan Coburger, Christian Rainer Wirtz, Michal Hlaváč

**Affiliations:** 1 Department of Neurosurgery, University of Ulm, Ludwig-Heilmeyerstr, 2, 89312 Günzburg, Germany; 2 Endokrinologiezentrum Ulm, Bahnhofplatz 7, 89073 Ulm, Germany; 3 Department of Neurosurgery, University of Ulm, Albert-Einstein-Allee 23, 89081 Ulm, Germany; 4 Institute for Epidemiology and Medical Biometry, University of Ulm, Schwabstraße 13, 89075 Ulm, Germany

**Keywords:** Intraoperative MRI, Pituitary adenoma, Knosp 0–2, Additional resection, Gross total resection

## Abstract

**Introduction:**

Intraoperative magnetic resonance imaging (iMRI) improves the intraoperative detection of adenoma remnants in transsphenoidal surgery. iMRI might be redundant in endoscopic pituitary surgery in non-invasive tumors (Knosp 0–2) due to a superior visualization of anatomical structures in the periphery of the sella turcica compared to the microscopic technique. We identified the anatomical location of tumor remnants in iMRI and evaluated risk factors for secondary resection after iMRI and hereby selected patients with pituitary adenomas who may benefit from iMRI-assisted resection.

**Methods:**

We conducted a retrospective monocenter study of patients who underwent iMRI-assisted transsphenoidal surgical resection of pituitary adenomas at our department between 2012 and 2020. A total number of 190 consecutive iMRI-assisted transsphenoidal surgeries of pituitary adenomas graded as Knosp 0–2 were selected for analysis. Exclusion criteria were missing iMRI availability or pathologies other than adenomas. Of these 190 cases, 46.3% (*N* = 88) were treated with microscopic, 48.4% (*N* = 92) with endoscopic, and 5.3% (*N* = 10) with endoscopic-assisted technique. Volumetric measurement of preoperative, intraoperative, and postoperative tumor extension was performed. Demographic data, tumor characteristics, and MRI features were evaluated. Additionally, analysis of adenoma remnants identified by iMRI was performed.

**Results:**

An additional resection after iMRI was performed in 16.3% (*N* = 31). iMRI helped to reach gross total resection (GTR) in 83.9% (26/31) of these cases. False-positive resection was found in 1 patient (0.5%). Multivariable logistic analysis identified tumor volume (OR = 1.2, *p* = 0.007) recurrence (OR = 11.3, *p* = 0.002) and microscopic technique (OR = 2.8, *p* = 0.029) as independent risk factors for additional resection. Simultaneously, the endoscopic technique was significantly associated with GTR as evaluated by iMRI (OR = 2.8, *p* = 0.011) and postoperative MRI (OR = 5.8, *p* = 0.027). The detailed analysis of adenoma remnants on iMRI revealed the suprasellar location in a diaphragm fold, penetrating tumor above the diaphragm, or undetected invasion of cavernous sinus as well as in case of microscopic resection tumor location outside the line of sight as the main reasons for incomplete resections.

**Conclusion:**

Tumor volume, recurrence, and microscopic technique were identified as independent predictors for additional resection in patients with Knosp 0–2 adenomas. iMRI might increase the extent of resection (EOR) safely even after the endoscopic visualization of the sella with very low risk for false-positive findings. Remnants of tumors hidden within the diaphragmic folds, intrathecally, or behind the infiltrated wall of cavernous sinus not recognized on preoperative MRI were the most common findings in iMRI.

## Introduction

Improvement of surgical techniques and the implementation of endoscopic transsphenoidal approach results nowadays in very high rates of gross total resections (GTR) [6, 7, 13]. Nevertheless, even small tumor remnants might result in progressive recurrence requiring additional surgery or adjuvant treatment [6, 17]. Intraoperative magnetic resonance imaging (iMRI) has been shown to have a complementary effect on both endoscopic and microscopic surgical techniques and might increase the extent of resection in pituitary adenomas [9, 12, 13, 17]. The use of an endoscope with improved visualization of the sella leads to the question, whether it is beneficial to use iMRI in the resection of adenomas graded as Knosp 0–2, and if so, which patients might benefit most from this additional imaging. The predictive factors for additional resection after iMRI have not yet been well defined. We have analyzed the location and characteristics of tumor remnants identified during surgery by iMRI after endoscopic and microscopic pituitary adenomas Knosp 0–2 and evaluated the potential risk factors for additional resection in this patient cohort.

## Methods

We conducted a retrospective monocenter data analysis of patients treated by iMRI-assisted transsphenoidal surgery at our department between 2012 and 2020. All patients without iMRI or with a histopathological diagnosis other than pituitary adenoma were excluded from the study. Only patients graded as Knosp 0–2 were involved in the evaluation. iMRI features, tumor remnants, volumetric analysis, and Knosp grading were performed with neuroradiologic assistance in cooperation with the department of neuroradiology at our department. A total number of 190 cases met the inclusion criteria. The detailed volumetric analysis was performed as described in our previous publication and a detailed evaluation of intraoperative remnants was done in cooperation with the department of neuroradiology [6, 9]. These findings were confirmed by a separate histopathological examination of tissue samples removed after iMRI. The one case of false-positive resection was not included in further analysis. The following categories for intraoperative adenoma remnants were assessed: infiltration of cavernous sinus wall, suprasellar extension, location of the tumor out of the microscopic line of sight, invasion in the recesses of the sphenoid sinus, and suprasellar intrathecal invasion with perforation of the diaphragma sellae. Furthermore, we separately evaluated the location of residual tumors, if GTR was not achieved.

A postoperative assessment of the clinical and endocrine status as well as MRIs was performed 3 months and 15 months after surgery. Thereafter, serial MRI scanning was performed on yearly basis or every 2 years depending on radiologic or biochemical evidence of the tumor. Basic demographic data such as age, sex, and basic adenoma characteristics such as recurrent adenoma, subtype of adenoma, or apoplectic form were noted.

The pituitary function was evaluated in cooperation with endocrinologists in a multidisciplinary approach before surgery, 4–6 weeks and 3–6 months after surgery. The endocrine evaluation was described in our previous publication [5]. A new permanent diabetes insipidus was diagnosed, if medical therapy was needed during follow-up.

Furthermore, visual deficits due to tumor-induced compression of the optic apparatus were documented before surgery. Visual field testing was repeated regularly after surgery. Improvement, stable state, and worsening of visual alterations were evaluated.

### Intraoperative MRI setup

An intraoperative 1.5 T MRI Espree scanner is available (Siemens AG, Erlangen, Germany) at our department as a one-room solution since October 2008. The analysis of intraoperative residual tumor was performed on thin 2-mm slice high-resolution coronal and sagittal T2 and gadolinium-enhanced T1 images using the Brainlab Elements software (BrainLab, München, Germany). A complete scan with contrast-enhanced T1 sequences was repeated at surgeons’ discretion. Postoperative MRI was performed 3 months after surgery and every year or every 2 years thereafter. Pre- and postoperative MRI images were acquired either with the intraoperative scanner or with a 1.5 T MRI Symphony system (Siemens AG, Erlangen, Germany).

### Surgical procedure

Endoscopic or microscopic approaches were conducted based on the surgeons’ preference. An endoscopic transsphenoidal binostril approach was performed with rigid 0°, 30°, and 45° Hopkins endoscopes using a 4 hands technique. In cases of invasive adenomas with skull base infiltration, extended endoscopic approaches in cooperation with ENT (ear nose throat) surgeons were carried out. The microscopic approach was performed in the standard direct transnasal transsphenoidal route. Endoscopic assistance during resection or inspection of the resection cavity was performed in selected cases and was deemed a separate category. Intraoperative navigation (BrainLab) was used routinely in all surgeries to define the resection borders within the sella. After bony decompression of the sella and durotomy, pituitary adenoma was removed. iMRI followed after tumor removal and thorough inspection of the resection cavity. Intraoperative scanning prolonged the time of the surgery for approximately 45 min.

The reconstruction of the skull base was performed with fibrin coated sponge in the case of a small or no intraoperative cerebrospinal fluid (CSF) leak. Large defects were sealed using a multilayer technique with abdominal subcutaneous fat graft and fibrin coated sponge or a nasoseptal flap according to our internal protocol. All surgeries were accomplished by authors specialized in the field of transsphenoidal surgery (AP, CRW, and MH). The aim was always to achieve maximal tumor removal before iMRI.

### Surgical complications

Postoperative meningitis was assumed when antibiotic treatment was initiated because of typical clinical signs of meningeal inflammation, even if no pathogen was isolated. CSF fistula was considered a complication if a lumbar drain not related to surgical procedure or revision surgery was necessary. Furthermore, intra- and postoperative bleeding, thromboembolic complications, and permanent or transient new neurological deficits were included. Hypopituitarism and diabetes insipidus were evaluated separately as described above.

### Data analysis

Statistical analysis was performed using SPSS 26.0 (Lead Technologies, INC, Charlotte, USA). Descriptive statistics as well as Mann–Whitney *U* and Fisher exact tests were used for the analysis. Univariate and multivariable logistic regression analyses for additional resection were calculated. Influencing variables were age, initial tumor volume, sex, recurrent surgery, and surgical technique. All variables which achieved significance (*p* < 0.05) in univariate analysis were included in multivariable analysis. Progression-free survival and Kaplan–Meier curve comparing patients after GTR and after subtotal resection with log-rank test was performed. ROC analysis and the Youden index were used for the calculation of cutoff values.

The study was conducted according to the international Declaration of Helsinki. The approval of the local ethics committee has been obtained.

## Results

### Demographic data and extent of resection

Out of 190 patients included in the analysis, 46.3% (*N* = 88) were treated with microscopic 48.4% (*N* = 92) with an endoscopic and 5.3% (*N* = 10) with an endoscopic-assisted technique. Basic demographic data are summarized in Table 1. The most common histopathological subtype was non-functioning adenoma (71.6%, *N* = 136), followed by GH-producing adenoma (16.3%, *N* = 31) and ACTH producing adenoma (8.9%, *N* = 17). Apoplectic adenoma was identified in 21 cases (11.1%). Median tumor volume was 3.08 cm^3^ (range 0.01–18.53 cm^3^).

**Table 1:** Patients’ and adenoma characteristics

	Total
*n*	190
Age (mean)	55
male ratio	55.8% (106)
Median tumor volume (cm^3^)	3.08
Recurrent adenoma	6.3% (12)
Apoplectic adenoma	11.1% (21)
Additional resection after iMRI	16.3% (31)
GTR	93.7% (178)

Additional resection after iMRI was performed in 16.3% (*N* = 31). iMRI helped to reach GTR in 83.9% (26/31) of these cases. False-positive resection was found in 1 patient (0.5%) and lead to transient diabetes insipidus. Stratified according to surgical technique, in the patient cohort treated by microscopic technique 22.7% (*N* = 20/88) underwent additional resection, while with endoscopic technique additional resection was performed in 9.8% (*N* = 9/92) and with endoscopic-assisted surgery in 20% (2/10). Overall GTR was achieved in 93.7% (*N* = 178). Final GTR was significantly more common with the endoscopic technique (*p* = 0.027, OR 5.8 CI95% 1.2–27.1, 97.7%, *N* = 88/90 vs 88.6%, *N* = 78/88). We confirmed significant advantage in GTR rates for endoscopic technique with iMRI before potential additional resection (*p* = 0.011, OR 2.8 CI 95% 1.2–5.9, 88%, *N* = 81/92 vs 72.7%, *N* = 64/88). Tumor volume (*p* = 0.705), age (*p* = 0.872), and recurrence (*p* = 0.063) showed no significant difference in regard to final GTR.

### Risk factors for additional intraoperative resection

Tumor volume, recurrence, and surgical technique showed a significant impact on additional resection in both univariate and multivariate analysis (Table 2). Age, sex, and adenoma subtype did not reach statistical significance (Table 2). Adenoma cutoff volume for higher risk of additional resection was 2.6 cm^3^ according to ROC analysis (AUC 0.641). Additional resection did not result in a significant increase of new hypopituitarism (*p* = 0.140), which was noted in 36 (18.9%) of all cases and was significantly more common using the microsurgical technique (*p* = 0.018). A new deficiency of at least one axis was seen in 23 (26.1%) patients treated microscopically, 10 (10.9%) patients treated endoscopically, and 3 (30%) patients with endoscopic-assisted surgery. Permanent diabetes insipidus was diagnosed in 6 patients (3.2%). Out of the total, 2 (1.1%) were treated by microscopic, 3 (1.6%) by endoscopic, and 1 (0.5%) by endoscopic-assisted technique.

**Table 2:** Univariate and multivariable analysis of factors with possible impact on additional resection after intraoperative MRI (*HR*, hazard ratio; *CI*, confidence interval)

	Univariate analysis			Multivariable analysis		
	HR	95% CI	*p*	HR	95% CI	*p*
Age	1.0	0.98–1.02	0.996			
Sex	1.9	0.8–4.5	0.132			
Recurrent surgery	6.1	1.6–22.6	**0.007**	11.3	2.5–51.3	**0.002**
Surgical technique	2.7	1.2–6.3	**0.021**	2.8	1.1–7.0	**0.029**
Tumor volume	1.1	1.0–1.2	**0.025**	1.2	1.0–1.3	**0.007**

### Intraoperative tumor remnants

**Fig. 1:**
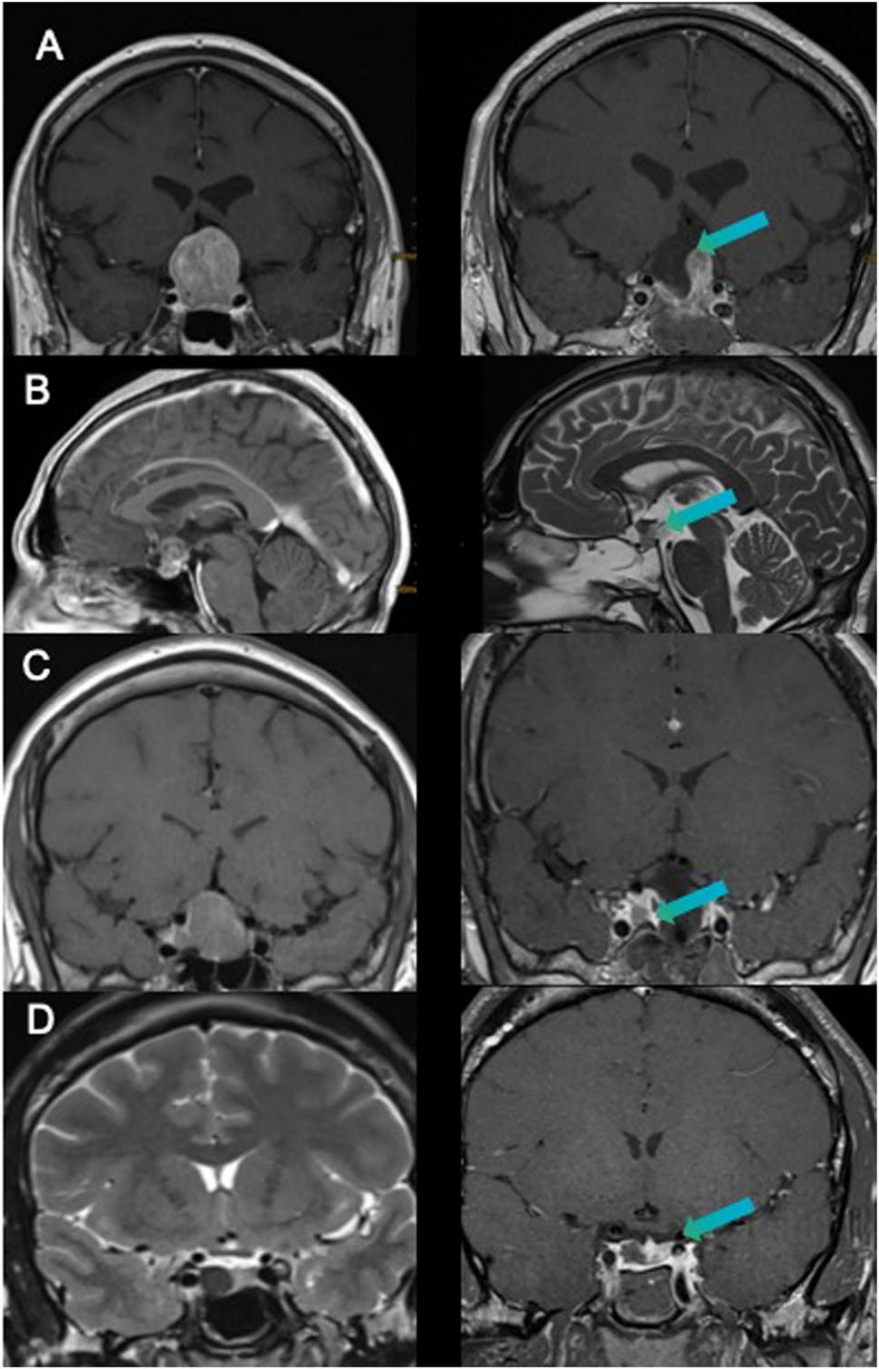
Different location of residual adenomas visible in intraoperative magnetic resonance imaging (**A** adenoma remnant in diaphragm fold, **B** intrathecal invasion through the diaphragm, **C** invasion in the cavernous sinus, **D** dumbbell-shaped adenoma not visible in preoperative images

The detailed analysis of intraoperative tumor remnants showed that the most common location of tumor remnant was inside the cavernous sinus (*N* = 13), the second most common location was a suprasellar extension in a diaphragm fold (*N* = 11). Further anatomical locations leading to subtotal resection were suprasellar intrathecal location (*N* = 2), invasion into the sphenoid sinus in the case of microscopic technique (*N* = 2), and infiltration of clivus (*N* = 1). In one case, a unique dumbbell formed adenoma (GH-producing adenoma) lead to incomplete resection (*N* = 1) and in another case the small remnant was firm and solid and attached to the pituitary gland (*N* = 1). Examples of tumor remnant locations are depicted in Fig. 1. If we analyzed tumor remnants after endoscopic resection, we found in 4 cases infiltration of the cavernous sinus, in 3 cases remnant within suprasellar diaphragm fold, in 2 cases intrathecal suprasellar extension, and in one case clivus infiltration. Median tumor volume evaluated on iMRI was 0.14 cm^3^ for microscopic and 0.096 cm^3^ for endoscopic technique (*p* = 0.04).

### Postoperative tumor remnants

Tumor remnant despite iMRI and further resection was found in 5 patients. Out of them, only one patient was treated endoscopically. In this case, a tumor remnant located within the clivus was found in a follow-up MRI scan. The remaining four patients underwent microscopic resection and in 2 of them tumor remnants were infiltrating the cavernous sinus, and in the other 2 cases, small residual tumors were located intradurally and out of sight of the microscope.

Furthermore, in 7 cases, GTR was not achieved according to postoperative evaluation, but no further resection was performed after iMRI. In these cases, tumor remnants were identified in the postoperative imaging 1 year after surgery or if biochemical remission in hormonally active tumors was not achieved despite negative MRI findings. Out of this group, 6 patients were treated microscopically and one endoscopically. In 4 patients, tumor remnants were found within the cavernous sinus; in one case, the tumor remnant was adherent to the pituitary stalk intradurally. In two cases treated for acromegaly, patients showed a persistence of the disease despite no obvious remnant in the iMRI. One of these 2 patients was treated endoscopically.

### Progression-free survival

**Fig. 2:**
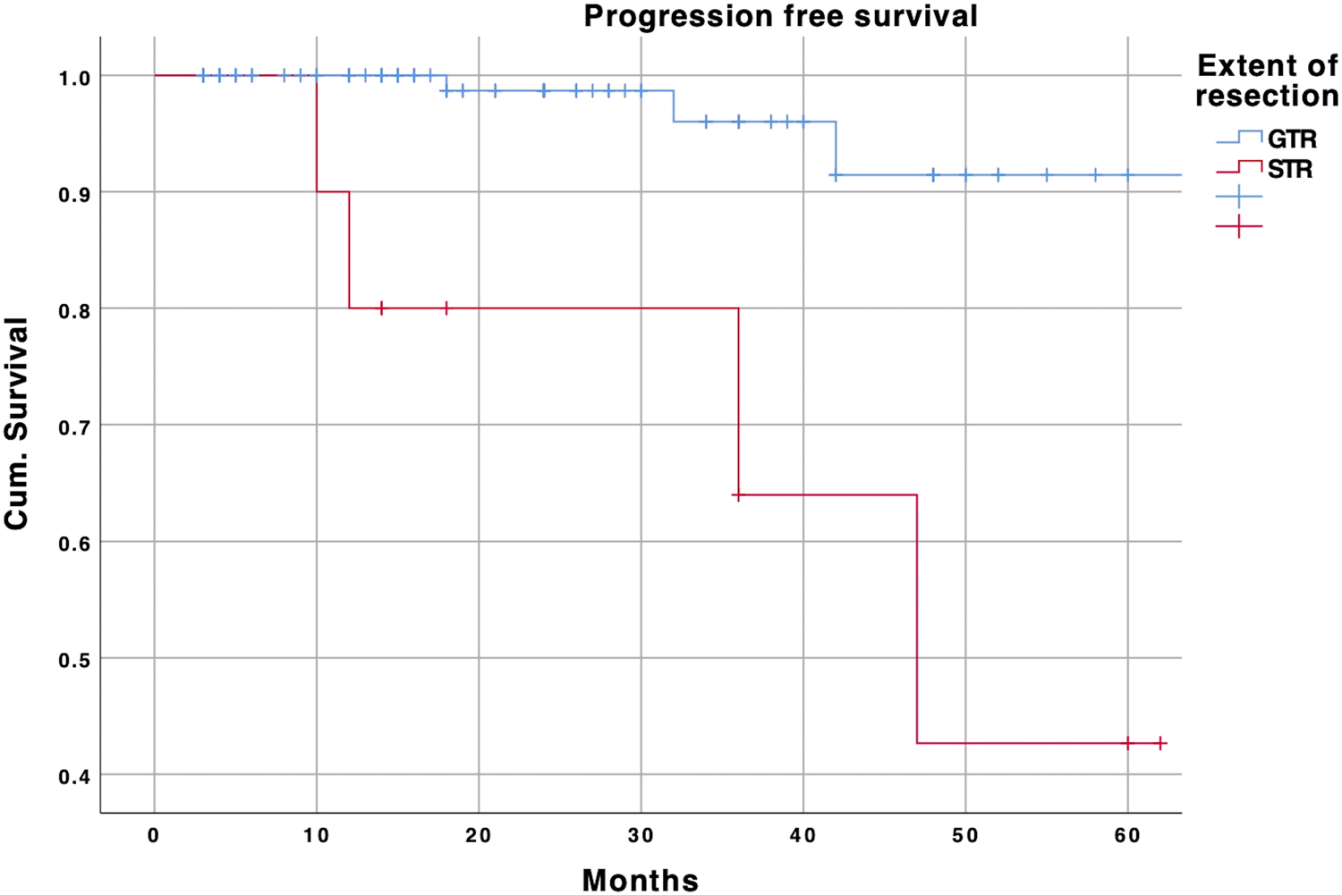
Kaplan–Meier curve depicting longer progression-free survival of patients after gross total resection

The median follow-up was 47 months. GTR significantly increased PFS if compared to STR (*p* < 0.001, Fig. 2).

### Functional adenomas

In regard to functional adenomas, biochemical remission has been achieved in 79.5% of cases (*N* = 35/44). Additional resection was performed in 7 cases and was not associated with a higher chance of biochemical remission (*p* = 0.619).

### Complications

Surgical revision was necessary in 6.3% (*N* = 12 cases) and was mostly performed due to CSF leak (4.2%, *N* = 8). In 2 patients (1.1%), revision surgery was performed because of epistaxis and in 2 (1.1%) patients due to intra- and suprasellar bleeding. Visual deterioration was noted in one of these bleedings that needed surgical revision. Meningitis was diagnosed in 2 patients (1.1%). We found no significant association between surgical technique and complications (*p* = 0.976).

## Discussion

Various studies have demonstrated an increase in the extent of resection by the use of iMRI in different intracranial pathologies including pituitary adenoma [4, 5, 8–10, 13, 17]. The evolution of surgical technique and increasing use of endoscopy in pituitary surgery leads to the question, if iMRI really helps in the detection of tumor remnants in the case of less invasive adenomas Knosp 0–2, in which resection rates even without iMRI are supposed to be very high [2, 12]. To address this, we have evaluated patients treated with the endoscopic and microscopic iMRI-assisted transsphenoidal technique with the focus on detailed characteristics of tumor remnants in the iMRI images. Microscopic technique, tumor volume, and tumor recurrence were confirmed as independent predictors for additional tumor resection following iMRI. Nevertheless, in almost 10% of cases treated with endoscopic technique, additional resection was also performed. Tumor remnants were mostly located in a suprasellar diaphragm fold, infiltrating the cavernous sinus, or were located intrathecally perforating the diaphragm. The cavernous sinus invasion was not noted on the preoperative images in these cases.

As reported in the systemic review published by Soneru et al., endoscopic technique and iMRI may have a complementary effect on the extent of resection (EOR) of pituitary macroadenomas [15]. In a more detailed evaluation of our iMRI data, we focused on further identification and selection of macroadenomas which might benefit from the combination of both techniques. The risk of detectable tumor remnant seems particularly increased in cases when the tumor is large and the diaphragm is stretched, so that the development of diaphragmatic folds can be anticipated; an intrathecal tumor extension cannot be ruled out or the tumor has a large interface with the cavernous sinus even without obvious invasion in the preoperative images. Since most of the surgically treated tumors exhibit at least one of the mentioned properties, we can encourage the routine use of iMRI in pituitary patients treated with the endoscope. Based on our experience, iMRI helps to increase the safety in these cases and might even in some cases help to avoid unnecessarily supradiaphragmatic exploration which represents an additional risk for CSF leakage after surgery. As a consequence of our results, we have abandoned microscopic resection without iMRI. If an MRI scanner is not available, we perform endoscopic resection and large adenomas or recurrences are primarily planned for iMRT-assisted resection.

The risk of false-positive findings and the consecutive more aggressive exploration which may lead to aggravation of endocrine deficits is the matter of many discussions. In our cohort, we have analyzed all histopathological findings before and after iMRI separately. We have identified only one patient with a false-positive iMRI which resulted in a transient diabetes insipidus after more aggressive exploration of the sella. This, however, resolved completely after 3 months. Based on our data, the incidence of a false-positive iMRI finding seems to be very low. Our results are in line with the conclusions published by Bellut et al. [2]. In this study, the low-risk profile of iMRI and the satisfactory endocrine outcome have been reported after the transsphenoidal pituitary surgery.

As presented by Sylvester et al. and confirmed by our study group, the increase in EOR is beneficial for longer PFS and might reduce the need for additional treatments [17]. Based on that, even if iMRI is surely not mandatory in endoscopic resection of Knosp 0–2 adenomas, it might nonetheless safely increase EOR and thereby improve PFS. It also might diminish the need for further surgery or adjuvant treatment especially in large adenomas with suprasellar growth or if adherence and potential infiltration of the cavernous sinus are suspected. Furthermore, it is important to point out that the resection of the medial wall of the cavernous sinus and an extensive intradural exploration is more easily performed with the endoscopic technique and limited with the microscopic approach.

Dalapiazza et al. compared the EOR between the endoscopic and microscopic techniques in Knosp 0–2 pituitary adenomas and found no significant difference between these techniques [3]. Interestingly, tumor remnants were identified on follow-up MRI in 10% after microscopic and in 9% of the cases after endoscopic resections. These findings correspond with our results before iMRI and additional resection underlining the previous conclusion. A meta-analysis published by Almutairi et al. showed the potential benefit of the endoscopic technique for GTR compared to microscopic transsphenoidal resection [1]. However, based on the heterogeneity of studies, a clear superiority has not been demonstrated so far. EOR is generally very high with either surgical techniques so that differences are small and difficult to prove. Nevertheless, there are ever more factors in favor of the endoscopic technique. The extended skull base approaches are the domain of endoscopy allowing the resection of suprasellar compartments of tumors with firm consistency that do not descend into the sella [11]. These remnants tend to undergo hemorrhagic transformation postoperatively and lead to visual deficits. Furthermore, the only prospective study which tried to compare the endoscopic with the microscopic technique had to be stopped since not enough surgeons using the microscopic technique were available in the participating centers [7]. Additionally, there are other factors which are more in the focus of ongoing studies such as the endocrine outcome and quality of life. The literature suggests that the endoscopic technique is positively associated with both. Staartjes et al. reported the benefit of intraoperative 3 T MRI in the resection of less invasive adenomas [16]. The main argument was the potential resectability of small intra- or suprasellar remnants which have a relevant impact on further recurrence. These data are in line with our experience and our presented results. Furthermore, we have identified initial tumor volume, recurrence, and microsurgical technique as risk factors for additional resection after iMRI, so we might select the cohort of patients with pituitary adenoma which might benefit from iMRI-assisted transsphenoidal resection more precisely. The aforementioned study group has proposed a new scoring system based on the diameter between C4 segments of carotid artery and the largest diameter of adenoma depicted on coronal sequences defining 4 types of adenomas [14]. Tumors graded 2 and 3 have been shown to benefit from iMRI. All tumors with one exception of a dumbbell-shaped adenoma which needed additional resection in our study could be graded as 2 or 3 according to the mentioned Zurich pituitary score. Our data thus support this grading system. Together with independent predictors from our study, the sensitivity of remnant prediction could possibly be further enhanced, helping to select patients who would most probably benefit from iMRI.

Pseudocapsular resection has been established as the primary technique which surely helps to achieve GTR [18]. Nevertheless, in the case of large adenomas with intrathecal or intracavernous infiltration, it is not always possible to follow the capsule along the whole circumference. This makes the evaluation of GTR intraoperatively more difficult. iMRI can be helpful in this concern and identify hidden remnants thereby increasing the safety of the procedure [5]. The need for intentional perforation of the diaphragm in cases where intrathecal remnants are falsely assumed might be decreased.

The benefit of iMRI or endoscopic technique in regard to functional adenomas is yet to be confirmed [6, 15]. Due to early metabolic sequelae, these adenomas mostly do not reach large volumes which might be relevant in regard to iMRI. Frequently, these patients are treated in high-volume centers with a lot of experience with pituitary adenomas. Furthermore, functional adenomas represent a relatively small number of cases so that it would be difficult to show a significant benefit. However, as presented in our case of a dumbbell GH-producing adenoma, iMRI might still be helpful since it is able to increase EOR and hereby potentially the rate of biochemical remissions.

The main limitations of our study are its retrospective character and the absence of a control cohort treated without iMRI. The only reasons why patients do not undergo iMRI at our department during transsphenoidal surgery are the presence of MRI incompatible implants, a need for emergent surgery, or unexpected logistic or technical problems. We decided to exclude these patients from the analysis since this group is highly selected and involves only a small number of cases. Finally, since access to iMRI at our department is readily available, it is requested frequently and may result in a potential bias.

## Conclusion

According to our results, tumor volume, recurrence, and microscopic technique were independent predictors for additional resection in patients with Knosp 0–2 adenomas. Remnants of tumors hidden within the diaphragmic folds, intrathecally, or behind the infiltrated wall of the cavernous sinus not recognized on preoperative MRI were the most common findings in iMRI. Even with the endoscopic technique, iMRI might increase the EOR in adenomas Knosp 0–2.

## Data Availability

On demand.

## Code availability

Not available.

## References

[1] Almutairi RD, Muskens IS, Cote DJ, Dijkman MD, Kavouridis VK, Crocker E, Ghazawi K, Broekman MLD, Smith TR, Mekary RA, Zaidi HA (2018). Gross total resection of pituitary adenomas after endoscopic vs. microscopic transsphenoidal surgery: a meta-analysis. Acta Neurochir.

[2] Bellut D, Hlavica M, Muroi C, Woernle CM, Schmid C, Bernays RL (2012). Impact of intraoperative MRI-guided transsphenoidal surgery on endocrine function and hormone substitution therapy in patients with pituitary adenoma. Swiss Med Wkly.

[3] Dallapiazza R, Bond AE, Grober Y, Louis RG, Payne SC, Oldfield EH, Jane JA (2014). Retrospective analysis of a concurrent series of microscopic versus endoscopic transsphenoidal surgeries for Knosp Grades 0–2 nonfunctioning pituitary macroadenomas at a single institution. J Neurosurg.

[4] Hatiboglu MA, Weinberg JS, Suki D, Rao G, Prabhu SS, Shah K, Jackson E, Sawaya R (2009). Impact of intraoperative high-field magnetic resonance imaging guidance on glioma surgery. Neurosurgery.

[5] Hlavac M, Knoll A, Etzrodt-Walter G, Sommer F, Scheithauer M, Coburger J, Wirtz CR, Pala A (2019). Intraoperative MRI in transsphenoidal resection of invasive pituitary macroadenomas. Neurosurg Rev.

[6] Hlavac M, Knoll A, Mayer B, Braun M, Karpel-Massler G, Etzrodt-Walter G, Coburger J, Wirtz CR, Pala A (2020). Ten years’ experience with intraoperative MRI-assisted transsphenoidal pituitary surgery. Neurosurg Focus.

[7] Little AS, Kelly DF, White WL, Gardner PA, Fernandez-Miranda JC, Chicoine MR, Barkhoudarian G, Chandler JP, Prevedello DM, Liebelt BD, Sfondouris J, Mayberg MR, TRANSSPHER Study Group (2019). Results of a prospective multicenter controlled study comparing surgical outcomes of microscopic versus fully endoscopic transsphenoidal surgery for nonfunctioning pituitary adenomas: the Transsphenoidal Extent of Resection (TRANSSPHER) Study. J Neurosurg.

[8] Pala A, Coburger J, Scherer M, Ahmeti H, Roder C, Gessler F, Jungk C, Scheuerle A, Senft C, Tatagiba M, Synowitz M, Wirtz CR, Schmitz B, Unterberg AW 2019 To treat or not to treat? A retrospective multicenter assessment of survival in patients with IDH-mutant low-grade glioma based on adjuvant treatment J Neurosurg 1–810.3171/2019.4.JNS183395 10.3171/2019.4.JNS183395 31323633

[9] Pala A, Knoll A, Brand C, Etzrodt-Walter G, Coburger J, Wirtz CR, Hlavac M (2017). The value of intraoperative magnetic resonance imaging in endoscopic and microsurgical transsphenoidal pituitary adenoma resection. World Neurosurgery.

[10] Pala A, König R, Hlavac M, Wirtz CR, Coburger J (2015). Does the routine use of intraoperative MRI prolong progression free survival in low-grade glioma surgery? A retrospective study. Innov Neurosurg.

[11] Schaberg MR, Anand VK, Schwartz TH, Cobb W (2010). Microscopic versus endoscopic transnasal pituitary surgery. Curr Opin Otolaryngol Head Neck Surg.

[12] Schwartz TH, Stieg PE, Anand VK (2006) Endoscopic transsphenoidal pituitary surgery with intraoperative magnetic resonance imaging. Neurosurgery 58:ONS44–51– discussion ONS44–51. 10.1227/01.neu.0000193927.49862.b6 16479628

[13] Serra C, Burkhardt J-K, Esposito G, Bozinov O, Pangalu A, Valavanis A, Holzmann D, Schmid C, Regli L (2016). Pituitary surgery and volumetric assessment of extent of resection: a paradigm shift in the use of intraoperative magnetic resonance imaging. Neurosurg Focus.

[14] Serra C, Staartjes VE, Maldaner N, Muscas G, Akeret K, Holzmann D, Soyka MB, Schmid C, Regli L (2018). Predicting extent of resection in transsphenoidal surgery for pituitary adenoma. Acta Neurochir.

[15] Soneru CP, Riley CA, Hoffman K, Tabaee A, Schwartz TH (2019). Intra-operative MRI vs endoscopy in achieving gross total resection of pituitary adenomas: a systematic review. Acta Neurochir (Wien).

[16] Staartjes VE, Serra C, Maldaner N, Muscas G, Tschopp O, Soyka MB, Holzmann D, Regli L (2019). The Zurich Pituitary Score predicts utility of intraoperative high-field magnetic resonance imaging in transsphenoidal pituitary adenoma surgery. Acta Neurochir.

[17] Sylvester PT, Evans JA, Zipfel GJ, Chole RA, Uppaluri R, Haughey BH, Getz AE, Silverstein J, Rich KM, Kim AH, Dacey RG, Chicoine MR (2015). Combined high-field intraoperative magnetic resonance imaging and endoscopy increase extent of resection and progression-free survival for pituitary adenomas. Pituitary.

[18] Taylor DG, Jane JA, Oldfield EH (2017). Resection of pituitary macroadenomas via the pseudocapsule along the posterior tumor margin: a cohort study and technical note. J Neurosurg.

